# Role of extrathyroidal TSHR expression in adipocyte differentiation and its association with obesity

**DOI:** 10.1186/1476-511X-11-17

**Published:** 2012-01-30

**Authors:** Sumei Lu, Qingbo Guan, Yuantao Liu, Haibo Wang, Wei Xu, Xia Li, Yuchang Fu, Ling Gao, Jiajun Zhao, Xiangdong Wang

**Affiliations:** 1 The Institute of Cell Biology, Shandong University School of Medicine, Jinan, China, 250012; 2 Department of Endocrinology, Provincial Hospital affiliated to Shandong University, Jinan, China, 250021; 3 Department of Endocrinology, the Second Hospital of Shandong University, Jinan, China, 250033; 4 Department of Otolaryngology-Head and Neck Surgery, Provincial Hospital affiliated to Shandong University, Jinan, China, 250021; 5 Department of Nutrition Sciences, University of Alabama at Birmingham, AL35294, USA

**Keywords:** TSHR, obesity, adipogenesis

## Abstract

**Background:**

Obesity is known to be associated with higher risks of cardiovascular disease, metabolic syndrome, and diabetes mellitus. Thyroid-stimulating hormone (TSHR) is the receptor for thyroid-stimulating hormone (TSH, or thyrotropin), the key regulator of thyroid functions. The expression of TSHR, once considered to be limited to thyrocytes, has been so far detected in many extrathyroidal tissues including liver and fat. Previous studies have shown that TSHR expression is upregulated when preadipocytes differentiate into mature adipocytes, suggestive of a possible role of TSHR in adipogenesis. However, it remains unclear whether TSHR expression in adipocytes is implicated in the pathogenesis of obesity.

**Methods:**

In the present study, TSHR expression in adipose tissues from both mice and human was analyzed, and its association with obesity was evaluated.

**Results:**

We here showed that TSHR expression was increased at both mRNA and protein levels when 3T3-L1 preadipocytes were induced to differentiate. Knockdown of TSHR blocked the adipocyte differentiation of 3T3-L1 preadipocytes as evaluated by Oil-red-O staining for lipid accumulation and by RT-PCR analyses of PPAR-γ and ALBP mRNA expression. We generated obesity mice (C57/BL6) by high-fat diet feeding and found that the TSHR protein expression in visceral adipose tissues from obesity mice was significantly higher in comparison with the non-obesity control mice (*P *< 0.05). Finally, the TSHR expression in adipose tissues was determined in 120 patients. The results showed that TSHR expression in subcutaneous adipose tissue is correlated with BMI (body mass index).

**Conclusion:**

Taken together, these results suggested that TSHR is an important regulator of adipocyte differentiation. Dysregulated expression of TSHR in adipose tissues is associated with obesity, which may involve a mechanism of excess adipogenesis.

## Background

Obesity, a condition of body characterized by over accumulation of fat, is associated with increased risks of cardiovascular disease, metabolic syndrome, and diabetes mellitus [1,2]. However, the mechanism of obesity development is not fully understood, and has become a focus of extensive investigations. Generally, obesity occurs when energy intake by an individual exceeds the rate of energy expenditure. Adipose tissues are the major depots for energy storage. Obesity is known to be directly linked to the accumulation of body fat. At the cellular level, obesity can be considered as a hypertrophic disease resulted from an increase in the number or size of individual adipocytes. New fat cells come from a preexisting population of undifferentiated progenitor with high capability of proliferation and differentiation. So far, numerous factors or proteins have been implicated in the generation of new fat cells, including peroxisome proliferator-activated receptor γ (PPAR-γ, a member of the nuclear hormone receptor), CCAAT/enhancer binding protein (C/EBP, including C/EBP a, C/EBPβ and C/EBPσ), adipocyte lipid binding protein (ALBP) and adipocyte determination and differentiation factor 1 (ADD1) [3-5]. However, the relevance of those factors to obesity is unclear.

Thyroid-stimulating hormone (TSH) receptor gene (*TSHR*) encodes a transmembrane receptor which belongs to a subfamily of heptahelical G protein coupled receptors. In thyroid tissues, TSHR mediates the effects of TSH released from the anterior pituitary, and plays critical roles in thyroid development and function. Recent studies have demonstrated that TSHR is also present in non-thyroid tissues, such as hepatocyte [6] and adipocytes [7,8]. However, the physiological or pathological relevance of TSHR in these non-thyroid tissues is not completely known and is now under intensive investigations [9-12].

Several studies have suggested that TSHR expression in adipocytes may play an important role for adipogenesis. T. Onaya reported that the differentiation of rat preadipocytes was accompanied by an increased expression of TSHR [7]. Similar results were also observed in human orbital preadipocyte fibroblasts [13,14] and in mouse embryonic stem cells [15]. However, there have been reports showing TSHR expression was negatively correlated with adipogenesis [9]. The reason for the discrepancy in the above observations is unknown. In the present study, the association between adipocyte differentiation and TSHR expression was investigated in murine 3T3-L1 preadipocytes. In addition, we determined the TSHR expression in adipose tissues from both mice and human samples, and the relevance of TSHR expression in adipocytes to obesity was evaluated.

## Results

### Induction of differentiation in 3T3-L1 preadipocytes

**Figure 1:**
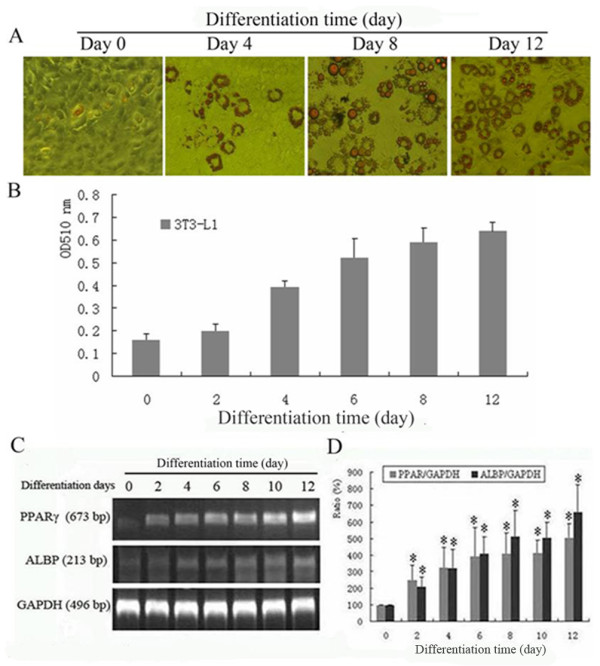
**Adipose differentiation of 3T3-L1 preadipocytes**. Adipose differentiation of 3T3-L1 preadipocytes was induced as described in Materials and Methods. (A) Adipose differentiation of 3T3-L1 preadipocytes evaluated by Oil-red-O staining of lipid droplets. (B) Quantitative analysis of the intracellular Oil-red-O by spectrophotometry. (C) PPAR-γ and ALBP mRNA expression at different stages of 3T3-L1 preadipoytes differentiation. (D) Quantitative analyses of PPAR-γ and ALBP mRNA levels at different stages of 3T3-L1 preadipoytes differentiation. Each experiment was repeated at least three times. *P < 0.05 *vs*. preadipocytes before induction.

3T3-L1 preadipocytes were induced to differentiate as described in **Materials and Methods**. The differentiation was evaluated by Oil-red-O staining for lipid accumulation and by RT-PCR analyses of PPAR-γ and ALBP mRNA expression. As shown in Figure 1 (A, B), lipid droplets could be detected by Oil-red-O staining as early as day-4 post differentiation induction, and peaked on day-12. To further verify the preadipocyte differentiation, the expression of PPAR-γ and ALBP, two essential regulators and markers for adipocyte differentiation, was analyzed by RT-PCR. As shown in Figure 1(C, D), the mRNA levels of PPAR-γ and ALBP were significantly increased on day-2 post induction of differentiation, and peaked on day-12 (*P *< 0.05).

### TSHR expression profile during the differentiation of 3T3-L1 preadipocytes

**Figure 2:**
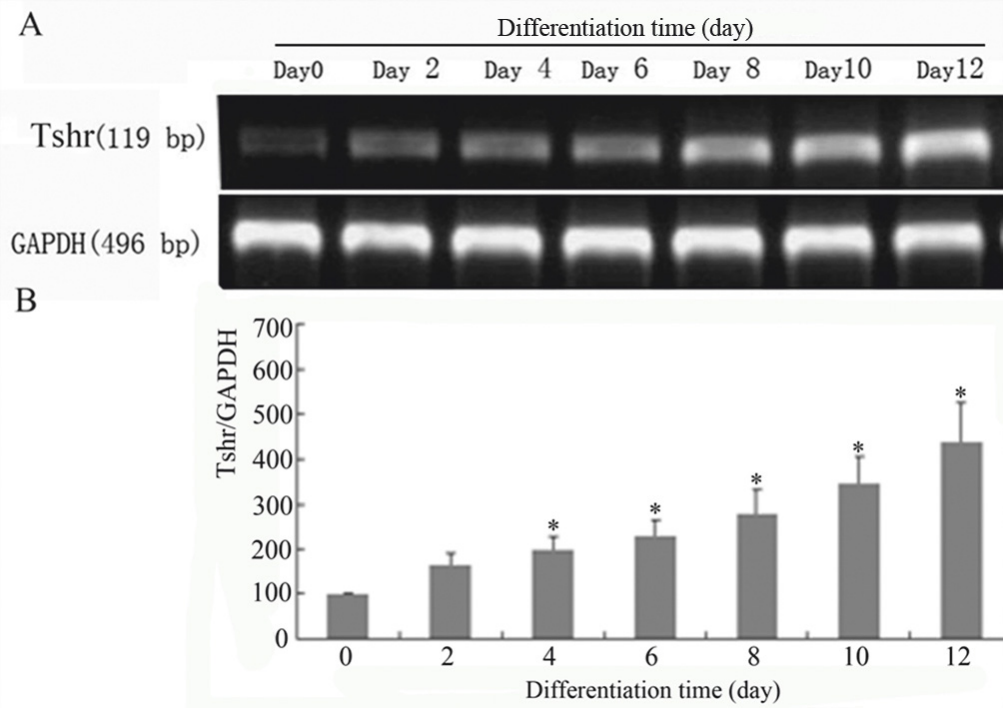
**TSHR mRNA expression profile during the adipose differentiation of 3T3-L1 preadipocytes**. (A) TSHR mRNA levels at different time points of differentitaiton determied by RT-PCR. (B) Quantitative analyses of TSHR mRNA levels determied by RT-PCR. The experiment was repeated three times. *P < 0.05 *vs*. preadipocytes before induction.

To get insight to the possible role of TSHR in adipogenesis, TSHR mRNA and protein levels during the differentiation of 3T3-L1 preadipocytes was analyzed. As shown in Figure 2, TSHR mRNA level was significantly increased 4 days after differentaition induction, and peaked on day-12.

**Figure 3:**
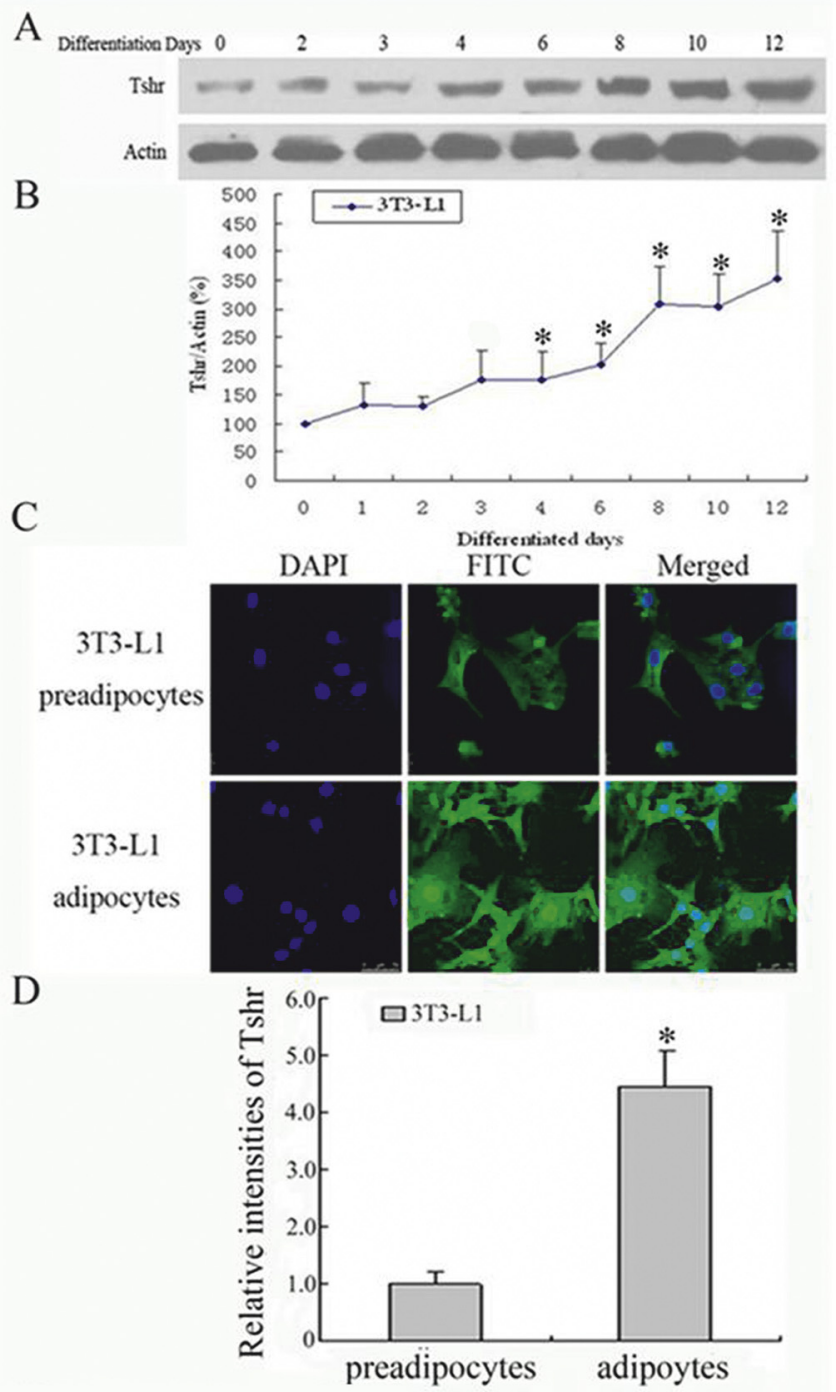
**TSHR protein expression profile during the adipose differentiation of 3T3-L1 preadipocytes**. (A) TSHR protein levels at different time points of differentitaiton determied by Western blot. (B) Quantitative analyses of TSHR mRNA levels determied by Western blot. (C) Representative photos of immuno-fluorescence staining for TSHR protein under Laser scanning confocal microscope. (D) Quantitative analysis of the fluorescence intensities of the differentiated adipocytes (12 days after induction) and undifferentiated 3T3-L1 preadipocytes. The average fluorescence intensity of the undifferentiated 3T3-L1 preadipocytes was designated as 1.

Consistent with the mRNA expression profile, the TSHR protein was showed to be significantly increased on day-4, and reached the peak on day-12 as determined by Western blot (Figure 3A/B). Additionaly, TSHR protein expression was analyzed by Laser scan confocal microscope following immuno-fluorescence staining. As shown in Figure 3C/D, TSHR protein expression was significantly higher in differentiated adipocytes (12 days post induction) than that of the undifferentiated 3T3-L1 preadipocytes.

### Influence of *Tshr *gene knockdown on 3T3-L1 preadipocytes differentiation

**Figure 4:**
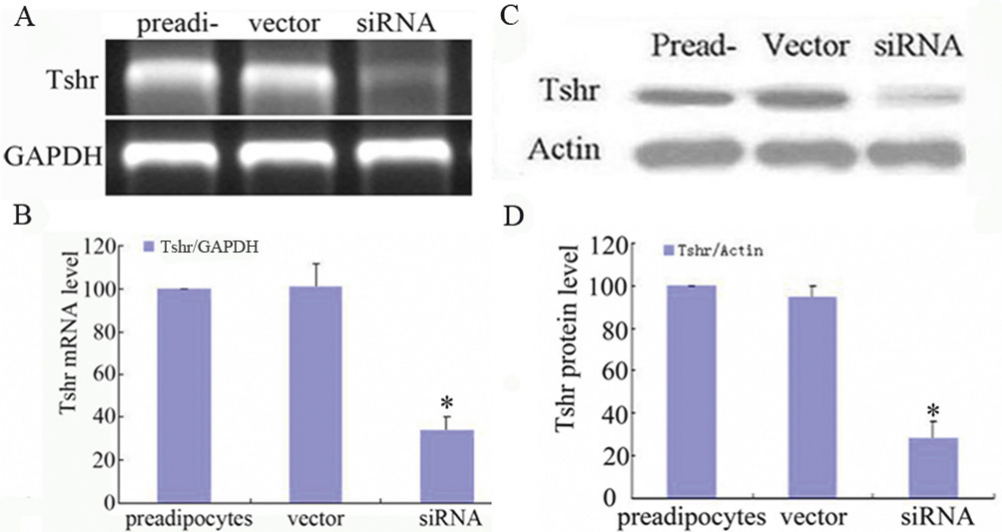
***Tshr *gene knockdown in 3T3-L1 preadipocytes**. 3T3-L1 preadipocytes were treated with siRNA or empty vector, respectively. After the selection of stable transfectants, TSHR mRNA and protein levels were determined by RT-PCR and Western blot.

**Figure 5:**
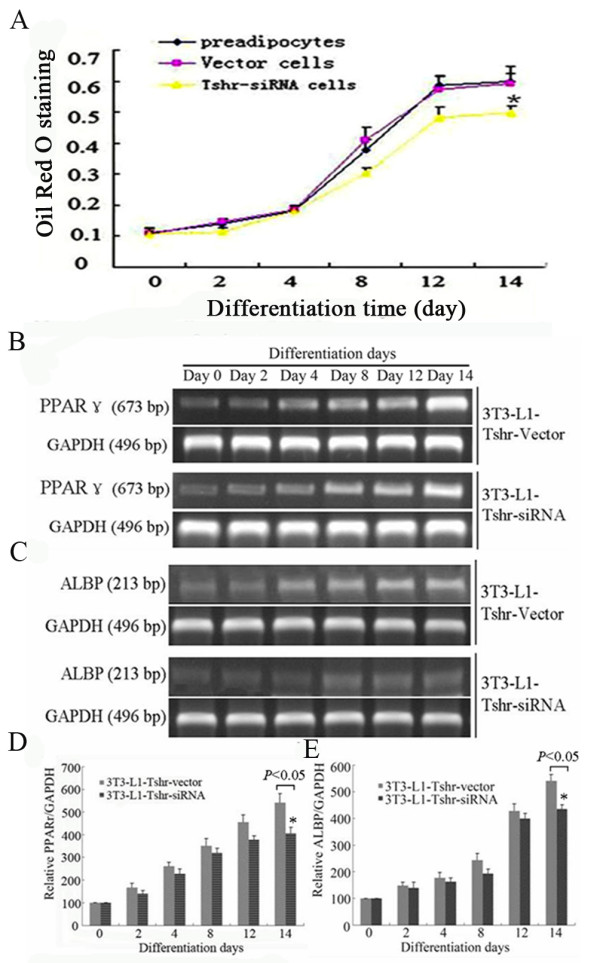
**Influence of *Tshr *gene knockdown on 3T3-L1 preadipocytes differentiation**. Stable transfected 3T3-L1 preadipocytes were induced to differentiate. Cell differentiation status was evaluated by analysis of endogenous lipid accumulation (A) and PPAR-γ and ALBP mRNA expression (B-E).

To get insight to the role of TSHR in adipose differentiation, *Tshr *gene expression was suppressed in 3T3-L1 preadipocytes by siRNA-mediated knock-down, and its influence on adipose differentiation was evaluated subsequently. As shown in Figure 4, siRNA treatment reduced the TSHR mRNA and protein expression by (60.73 ± 5.56)% and (71.33 ± 7.02)%, respectively, compared to vector treated control cells. When induced to differentiate, siRNA treated cells showed to have less intracellular lipid accumulation and lower levels of PPAR-γ and ALBP protein compared to vector treated control cells at each time point (Figure 5). These results indicated that TSHR-mediated signaling is necessary for adipose differentiation of 3T3-L1 preadipocytes.

### Increased TSHR protein expression in visceral adipose tissues from obese mice

**Figure 6:**
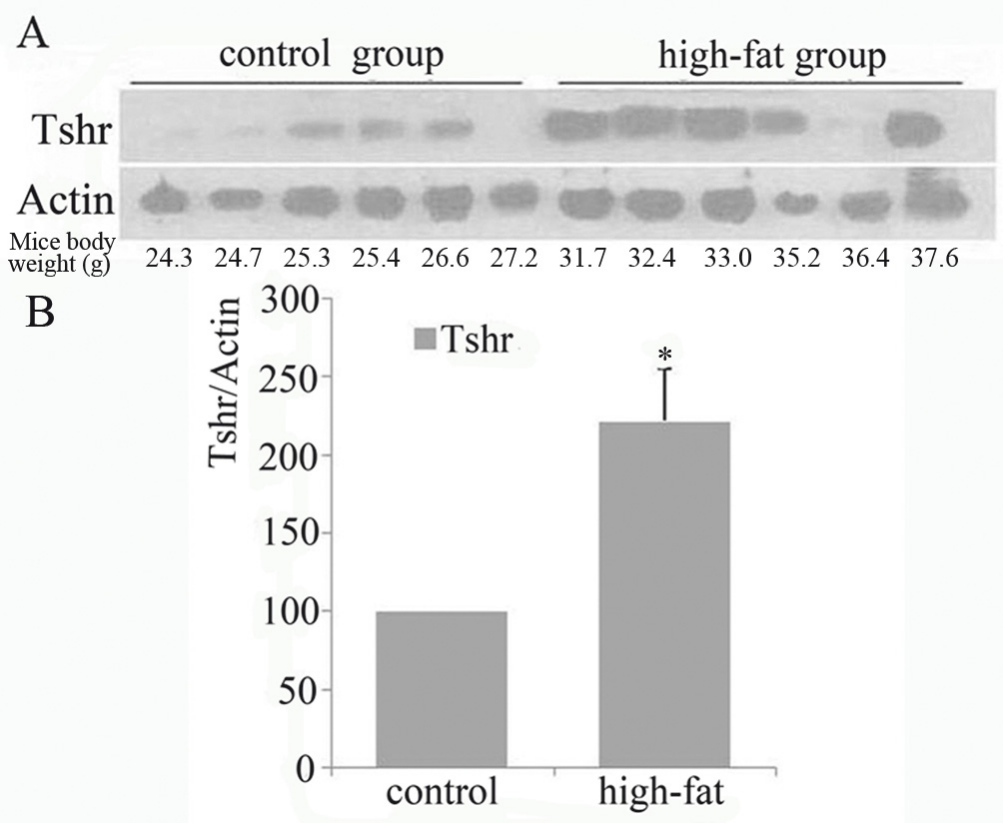
**Increased TSHR protein expression in visceral adipose tissues from diet-induced obese mice**. Visceral fat around the kidneys were extracted from both diet-induced obese mice and non-obese mice. TSHR protein level was determined by Western blot. (A) Representative result from Western blot analysis of TSHR protein expression. (B) Quantitative analysis of TSHR protein expression. The experiment was repeated three times.

Obesse C57/BL6 mice were induced with high-fat diet for 14 weeks, while control mice were fed with ordinary diet. At the end of experiments, the body weight, serum CHOL and GLU levels were increased in obese mice compared to the control mice (Table 1). In each group, TSHR protein expression in visceral adipose tissues showed to increase with increasing body weight. The average TSHR protein level in obese mice was significantly increased compared to control mice (Figure 6A/B).

**Table 1:** Metabolic parameters of control and High-fat diet mice.

*Variables*	*Basal diet-fed* *control mice*	*High fat diet-fed* *obese mice*	*P value*
n	10	6	
Body weight (g)	25.82 ± 1.05	37.66 ± 3.64	*P *< 0.05
AST (IU/L)	205.2 ± 7.33	204.8 ± 27.89	*P *> 0.05
TG (mmol/L)	0.97 ± 0.15	0.94 ± 0.11	*P *> 0.05
CHOL (mmol/L)	1.61 ± 0.31	3.41 ± 0.24 *	*P *< 0.05
GLU (mmol/L)	4.36 ± 0.83	8.55 ± 0.6 *	*P *< 0.05

Data are means ± SE. *P < 0.05, control vs. High-fat diet mice.

### Correlation between BMI and TSHR protein expression in human subcontaneous adipose tissues

**Figure 7:**
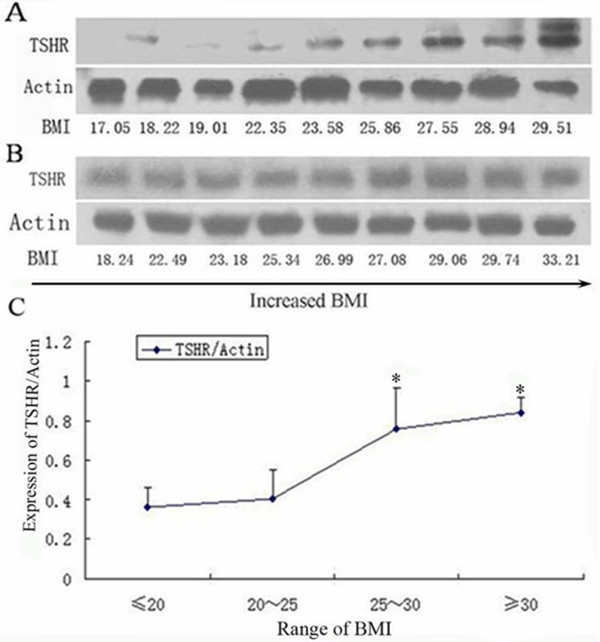
**Correlation between BMI and TSHR protein expression in human subcontaneous adipose tissues**. TSHR protein expression in human subcontaneous adipose tissues was analyzed by Western Blot. (A, B) Representative results from Western blot analysis of TSHR protein expression. (C) Quantitative analysis of TSHR protein expression based on BMI.

The TSHR protein expression in human adipose tissue samples was analyzed by Western blot. Our results showed that TSHR protein expression in human subcutaneous adipose tissues tend to increase with the increase in BMI (Figure 7).

## Discussion

Obesity is a common nutritional disorder, which is characterized by excess accumulation of body fat, and is known to be associated with increased susceptibility for cardiovascular disease, metabolic syndrome, and type 2 diabetes (T2DM) [16,17]. It has been suggested that obesity can be considered as a hypertrophic disease resulted from an increase in the number or the size of adipocytes. Adipocytes are derived from the fibroblast-like preadipoctes though cell differentiation, and dysregulated differentiation and proliferation of preadipocytes have been implicated in obesity development [18].

Thyroid-stimulating hormone (TSH) is a hormone released from the anterior pituitary, which is the main regulator of thyroid growth and function. The action of TSH is known to be mediated by thyroid-stimulating hormone receptor (TSHR) that belongs to the G protein coupled receptor family [19]. In addition to thyroid, TSHR has been proven to be expressed in fat cells. Several studies have suggested that TSHR expression in adipocytes may play an important role in adipogenesis. T. Onaya reported that the differentiation of rat preadipocytes was accompanied by an increased expression of TSHR [7]. Similar results were also observed in human orbital preadipocyte fibroblasts [13,14] and in mouse embryonic stem cells [15]. In the present study, we showed that TSHR expression increases with the adipose differentiation of 3T3-L1 preadipocytes. Our results are in agreement with previous observations [20-22]. Additionally, we showed that knockdown of TSHR delayed the adipocyte differentiation of 3T3-L1 preadipocytes, thus further confirming that TSHR is a possible regulator of adipogenesis.

To investigate the possible involvement of TSHR in the pathogenesis of obesity, we analyzed the TSHR expression profile in adipose tissues in mice. We established obesity model in C57/BL6 mice with high fat diet as described previously [23-25]. The successful establishment of obese model was confirmed by the significantly increased body weight and higher serum CHOL levels in obesity mice after 14 weeks of diet induction. Finally, TSHR protein expression in visceral adipose tissue was analyzed, and results showed that the average TSHR protein level in obese mice was higher compared to the non-obesity control mice. These findings suggest that diet-induced obesity is associated with an upregulation of TSHR expression in adipose tissue. However, we also found that TSHR protein expression was not increased in all obese mice. The mechanism for this discrepancy is not clear, which may reflect the complexity in obesity development.

In addition to animal experiment, TSHR expression in adipose tissues was further investigated in human samples. Subcutaneous adipose tissues from individuals with different BMI were used for TSHR determination. We found that the TSHR expression level was higher in people with BMI > 25 than that of those with BMI < 25.

Taken together, this study demonstrated that TSHR plays an important role in adipocyte differentiation. The TSHR expression in adipose tissues is associated with diet- induced obesity in mice and increases with increasing BMI in human. Our findings suggest that dysregulated TSHR expression might be implicated in obesity development under certain circumstances, which may involve a mechanism of excess adipogenesis.

## Materials and Methods

### Materials

The mouse embryo fibroblasts 3T3-L1 was obtained from the American Type Culture Collection (ATCC). Medium and serum were purchased from GIBCO (Invitrogen, USA). Anti-TSHR and anti-β-actin antibodies were purchased from Santa Cruz (Cambridge, UK). All primers used in this study were synthesized in Genomics Institute of HuaDa in Beijing. All other reagents were purchased from Sigma (St. Louis, MO, USA).

### Cell culture, preadipocyte differentiation induction

3T3-L1 preadipocytes were maintained in Dulbecco's Modified Eagle Medium (DMEM) supplemented with 5% calf serum, 100 U/mL penicillin and 100 mg streptomycin at 37°C in a humidified atmosphere composed of 95% air and 5% CO_2_. To induce differentiation, confluent preadipocytes were treated for 2 days with insulin (10 μM), isobutylmethylxanthine (IBMX) (0.5 mM), and dexamethasone (0.25 μM) in DMEM containing 10% FCS, followed by treatment for another 2 days with insulin (10 μM) alone in DMEM containing 10% FCS. Afterwards, cells were replenished with DMEM containing 10% FCS every other day. 10~12 days later, approximate 80% cells were differentiated into adipocytes.

### Oil-Red-O staining

After removal of culture medium, cells were washed three times with phosphate buffered saline (PBS) and fixed with 4% formaldehyde at room temperature for 10 min. After washing with PBS, cells were then stained with freshly diluted Oil-Red-O solution (6 parts of 0.1% Oil-Red-O in isopropyl alcohol and 4 parts of water) for 30min. Cells were then washed twice with 60% isopropyl alcohol for 1 min each, and washed once with PBS. Images were acquired under the inverted phase contrast microscope. For quantitative analysis, Oil-Red-O staining was dissolved with isopropyl alcohol and the optical density was measured at 510 nm by spectrophotometry. All experiments were performed in triplicate.

### Confocal fluorescence microscopy analysis

3T3-L1 preadipocytes were induced to differentiate for as described above. Cells were fixed in 4% formaldehyde for 15 min and blocked in PBS containing 3% BSA 30 min at 37°C in a humidified atmosphere. Subsequently, the cells were incubated for 16~18 h at 4°C with primary anti-TSHR antibody (1:200 dilution), followed by incubation with Fluorescein isothiocyanate (FITC)-labeled rabbit-anti-goat secondary antibody (1:200) for 30 min. After washing, cells were mounted with DAPI-containing mounting medium, and analyzed by confocal microscope (TCS SPE, Leica, Germany). For quantitative analysis of the TSHR expression, the fluorescence intensity of at least 25 randomly selected cells from the differentiated adipocytes (12days after induction) and undifferentiated 3T3-L1 preadipocytes was measured using the MetaMorph software (Universal Imaging Corporation, West Chester, PA).

### Knockdown of TSHR in 3T3-L1 preadipocytes

The siRNA targeting to TSHR was generated by using the lentivirus vector pLentiLox 3.7 according to the manufacturer's instruction. Briefly, the oligo sequences of the siRNA targeting to TSHR were as follows:

oligo-F: 5'-AACGTACAACAATGGATTTACTTTCAAGAGAAGTAAATCCATTGTTGTACTTTTTC-3'

oligo-R: 5'-TCGAGAAAAAGTACAACAATGGATTTACTTCTCTTGAAAGTAAATCCATTGTTGTACG

TT-3' The sequence targeting the TSHR gene-coding region were annealed and inserted into pLentiLox 3.7 vector between Xho I and Hpa I to generate the siRNA expression vector. The obtained plasmids (pLentiLox 3.7 siRNA-TSHR) were then used to generate lenti-virus encoding siRNA targeting to TSHR in 293T cells. pLentiLox 3.7 vector was used to generate non siRNA virus as control.

Plasmids (pLentiLox 3.7 siRNA-TSHR and pLentiLox 3.7) were transfected into 293T cells for packaging two viruses according to manufacture's protocol. 48 h later, the supernatants containing virus were used to infect 3T3-L1 when cells reached 80~90% confluence. 24 hours post infection, stable transfectants were selected. Cells with stable expression of pLentiLox 3.7 vector were used as controls.

### Obesity mice induction and analysis

Twenty-four adult male C57/BL6 mice aged 6 weeks were purchased from the Experimental Animal Center of Shandong University. Animal experiments were in accord to the 'Principles of laboratory animal care' established by the National Institutes of Health, and approved by the "Animal Care and Use Committee" of the Shandong University (number ECAESDUSM 2011037). Mice were maintained in a controlled environment at 25°C and 55% relative humidity environment with free access to tape water and food. After one week of adaptation, mice were randomly divided into two groups, the "high-fat group" and the "control group", and fed for 14 weeks with different rodent diets. The control diet contained 4% fat while high-fat diet contained 20% fat. Compositions of both control and high-fat diets were based on the basal diet, which was detected by Pony Testing International Group (Table 2). The "high-fat group" was fed with high-fat diet, while the "control group" was fed with control diet. The body weight of each animal was monitored once a week. The animals were sacrificed after deep anesthesia. Blood was collected in EDTA-coated tubes after fasting the mice for 12 hr, serum was separated by centrifugation (500×g, 4°C, 10 min). Serum cholesterol (CHOL), triglycerides (TG), aspartate transaminase (AST) and serum glucose (GLU) levels were measured using the Beckman DXC 800 analyzer (American). All these biochemical measurements were carried out in the Lab of Clinic Exam in Shandong Provincial Hospital. Visceral adipose tissues near the kidneys were collected and quickly frozen by immediate immersion in liquid nitrogen, and stored at -80°C for protein extraction.

**Table 2:** Contents of commercial basal diet detection.

*Ingredients*	*Limit*	*Test Content*
Moisture (%)	≤ 10	7.0
Protein (%)	≥ 18	20.1
Fat (%)	≥ 4	5.1
Fiber (%)	≤ 5	4.74
Ash (%)	≤ 8	6.67
Calcium (%)	1.0~1.8	1.19
Phosphor (%)	0.6~1.2	0.82
Arsenic (mg/kg)	≤ 0.7	0.36
Ab (mg/kg)	≤ 1.0	0.13
Cadmium (mg/kg)	≤ 0.2	0.098

Not detected Hg, BHC, DDT and Aflatoxin B1.

### Human fat sample collection

Adipose tissue samples were collected from patients with benign cernical lesions who underwent surgeries in the Department of Otolaryngology Division of Head and Neck Surgery in Shandong Provincial Hospital Affiliated to Shandong University. 120 patients were recruited in this study, which included 21 cases with obstructive sleep apnea-hyperpnoea syndrome (OSAHS), 51 cases with benign tumor of parapharyngeal space (BMPS), 30 cases with branchial cleft cyst (BCC) and 18 cases with thyroglossal tract cyst (TTC). The basic clinical characteristics of these patients were shown in Table 3. All these patients were euthyroid, and had no history of diabetes and no family history of obesity. These patients were divided in 4 groups based on their BMI (Body Mass Index, calculated as body weight (BW, kg) over squared height in meter): slim group (25 cases), BMI ≤ 20; normal group (33 cases), 20 < BMI < 25; overweight group (34 cases), 25 ≤ BMI < 30; obesity group (28 cases), BMI ≥ 30. Written informed consent was obtained from all patients before surgery. All patients were operated under general anesthesia. Adipose tissues were got from the subcutaneous areas in the necks. The study was approved by the Ethics Committees of Shandong University (number MECSDUMS 2011055).

**Table 3:** Human samples information collected from clinic.

*BMI*	*Number*	*Sex*		*benign cernical lesions*			
		
		male	female	OSAHS	BMPS	BCC	TTC
BMI ≤ 20	25	11	14	0	13	6	6
20 < BMI ≤ 25	39	18	21	1	18	15	5
25 < BMI ≤ 30	34	14	20	8	17	4	5
BMI > 30	22	14	8	12	3	5	2
Total number	120	57	63	21	51	30	18

### Adipose tissue pretreatment

Adipose tissues contain a large amount of lipids, which can disturb the processes for RT-PCR and Western-blot analyses. In the present study, we used a method to remove the majority of triglycerides from adipose tissues before further analyses. Briefly, about 50mg adipose tissues was cut into small pieces and homogenized with 100~200 μl RIPA (radio-immune precipitation buffer containing protein inhibitor) in a homogenizer. The lysate was transferred into 1.5ml Eppendorf tubes and mixed with ten times volume of pre-cooled acetone and shaken for 10 times. The mixture was centrifuged at 12000 g for 1 ~ 2 h at 4°C. Lipid droplets always appeared in the upper layer, and other components of cells always stayed in the bottom of the tube. The upper lipid droplets was removed and discarded. The same procedure can be repeated until lipids are effectively removed.

### RT-PCR

Total RNA was extracted using TRIZOl (Invitrogen) from adipocytes cells or primary adipose tissues. M-MuL V reverse transcription (Takara) was used for mRNA measurements. In brief, RT was performed by using the ExScript RT reagent kit (Takara Bio, Otsu, Shiga, Japan) in a final volume of 20 μL containing 1 μg total RNA, 4 μL 5×ExScript buffer, 1 μL deoxynucleotide triphosphate (dNTP, 10 μM) mixture, 1 μL Oligo (dT) primer, 0.5 μL ExScript RTase, 0.5 L RNase inhibitor, and RNase-free water to a volume of 20 μL. PCR was conducted according to the instructions of Takara Taq TM under the following conditions: pre-DNA denaturation at 95°C for 3 minutes; DNA denaturation at 95°C for 45 seconds; annealing for 40 seconds at temperatures: 53°C for Tshr, 55°C for ALBP or PPAR-γ, and 60°C for GAPDH; elongation is carried out at 72°C for 50 seconds, the total cycle number is 30. All experiments were performed in triplicate. The relative OD ratio was calculated using the NIH Image J software with GAPDH as an internal control. Primers used in the experiments were shown in Table 4.

**Table 4:** Sequence information on the primers used for RT-PCR.

Genes	Sequences	Product size (bp)	Annealing temperature (°C)	Gene Bank
Mouse *ALBP*	5'-GATGCCTTTGTGGGAACCTGG-3' 5'-TTCATCGAATTCCACGCCCAG-3'	213	55	NM_024406.2
Mouse *PPAR-γ*	5'-GACCACTCGCATTCCTTT-3' 5'-GGCATTGTGAGACATCCC-3'	459	55	NM_001127330.1
Mouse *Tshr*	5'-TCATTGCCTCTGTAGACCTG-3' 5'-TGATAACTCACTGGCGAAA-3'	119	53	NM_011648.5
GAPDH	5'-CAAGGTCATCCATGACAACTTTG-3' 5'-GTCCACCACCCTGTTGCTGTAG-3'	496	60	Complementary to human, mouse and rat GAPDH

### Western blot analysis

Samples were homogenized in RIPA lysis buffer containing protease and phosphatase inhibitors. Protein concentration was determined by Bradford method. Proteins (80 μg from each sample) were resolved on SDS-PAGE gels and transferred to a Hybond-P PVDF membrane. Subsequently, the membrane was blocked in 5% nonfat milk for 1 hour and incubated with anti-TSHR (1:1000) or anti-β-actin(1:2000) primary antibodies overnight at 4°C, followed by incubation with the peroxidase-conjugated anti-goat or anti-mouse secondary antibody (1:5000 dilution) for 1 h at room temperature. After washing with PBS, the bound primary antibody was visualized with the Chemiluminescence System (SuperSignal West Pico Chemiluminescent Substrate) (all from Pierce) and exposed to film. The relative intensity of TSHR to β-actin was analyzed with the Image J software.

### Statistical analysis

Data were presented as mean ± standard error of the mean (SEM). One-way analysis of variance (ANOVA) and *T *test were performed using the SPSS 13.0 software package. *P *< 0.05 was considered statistically significant.

## Competing interests

The authors declare that they have no competing interests.

## Authors' contributions

SML and QBG conducted research and analyzed data. YTL helped in statistical analysis. HBW and WX participated in human sample collections and detections. XL, YCF and LG helped in conducting research. XDW and JJZ designed the research and wrote paper. All authors read and approved the final manuscript.
